# SLAMF7/STAT6 Pathway Inhibits Innate Immune Response in Late-Stage Human *Acanthamoeba* Keratitis: A Comparative Transcriptome Analysis

**DOI:** 10.3390/microorganisms11020365

**Published:** 2023-02-01

**Authors:** Zhenyu Wei, Yuheng Zhang, Qiankun Chen, Xizhan Xu, Zhiqiang Pan, Zi-Bing Jin, Qingfeng Liang

**Affiliations:** Beijing Institute of Ophthalmology, Beijing Tongren Hospital, Capital Medical University, Beijing Key Laboratory of Ophthalmology and Visual Sciences, Beijing 100005, China

**Keywords:** *Acanthamoeba* keratitis, transcriptome analysis, immune status, differentially expressed genes, pathway enrichment

## Abstract

*Acanthamoeba* keratitis (AK) is a blinding corneal infection caused by the protozoan *Acanthamoeba*. The long-term course of AK suggests the host immunity could not kill *Acanthamoeba* rapidly. The immune status is still unclear in the late stage of AK. The comparative transcriptome analysis was made based on the bulk RNA sequencing of cornea tissues from AK patients and donors. Differentially expressed genes and enriched signaling pathways were calculated. CIBERSORT algorithm was used for immune infiltration analysis of cornea tissue between AK and normal controls. A total of 2668 differentially expressed genes, including 1477 upregulated genes and 1191 downregulated genes, were detected. Gene Ontology analysis revealed that the pathways were significantly enriched in leukocyte migration, regulation of T-cell activation, the external side of plasma membrane, collagen-containing extracellular matrix, immune receptor activity, and cytokine binding. The Kyoto Encyclopedia of Genes and Genomes pathway analysis showed that the pathways were significantly enriched in the cytokine–cytokine receptor interaction, hematopoietic cell lineage, and *Staphylococcus aureus* infection pathway. The immune infiltration profiles varied little between AK and normal controls. Compared with normal tissue, cornea tissue of AK contained a higher proportion of M0 macrophages and CD8 T cells, while resting memory CD4 T cells contributed to a relatively lower portion (*p* < 0.05). Finally, the expression levels of cell markers and SLAMF7/STAT6 pathway were confirmed by histopathology examinations, RT-qPCR, and Western blot.

## 1. Introduction

*Acanthamoeba* keratitis (AK) is a rare but potentially blinding corneal infection caused by the protozoan *Acanthamoeba*, an opportunistic free-living parasite. Contact lens (CL) wear and trauma become the most important risk factors for AK. With the increasing use of contact lenses around the world, the incidence of AK is obviously rising [1,2]. Carnt et al. reported 36–65 AK cases per year in the UK from 2010 to 2017, representing a three to five times increase in incidence compared to 2004–2009 (15–23 cases per year) [3]. Normally, the host immune system is capable of controlling this pathogen, while, under certain circumstances, for instance, wearing soft contact lens or orthokeratology [4], could render individuals more susceptible to *Acanthamoeba* infection. Because AK is frequently misdiagnosed as fungal or viral keratitis, as well as *Acanthamoeba* being able to differentiate into cysts inside host tissues, which resist multiple antiseptics, AK is difficult to treat and so may result in blindness and corneal scarring, even in immunocompetent people [5], as compared to *Acanthamoeba* encephalitis, which occurs in immunocompromised patients more often. Understanding the micro change in AK is essential to further explore more interventions for pathogen killing.

Classically, inherent immunity is the main way to kill protozoa. The activated macrophages polarized into the M1 phenotype, which produces nitric oxide synthase, reactive oxygen species, interleukin-1 β (IL-1β), and tumor necrosis factor-alpha (TNF-α) to defend against infection [6]. Meanwhile, macrophages can also be alternatively polarized into the M2 phenotype, which produces arginase-1 to control excessive inflammation and promote wound healing [6,7]. However, the histopathology showed the absence of inflammatory infiltrates in the deep stroma of the cornea with *Acanthamoeba* [8]. Even the zone containing amoebic cysts could be without noticeable inflammation [9]. Although neutrophils are promptly recruited and visible around *Acanthamoeba* trophozoites in the early inflammatory response of innate immunity [10,11], the histopathology suggested the disturbed immune response in AK, especially in the late stage, in which *Acanthamoeba* are mainly present in the cornea as cysts. RNA sequencing could allow for the deep and efficient probing of the transcriptome, which may provide some clues about host immune reactivity to the *Acanthamoeba*. The immune status and the roles of macrophages in the late stage of AK could be furtherly investigated, based on transcriptome sequencing technologies.

Signaling lymphocyte activation molecule (SLAM) family receptors are expressed on different types of hematopoietic cells and play an important role in immune regulation in health and disease. SLAM family-7 (SLAMF7), an immunomodulatory transmembrane receptor that is highly and uniformly expressed, is expressed on macrophages, natural killer cells, and other immune cells [10]. Previously, SLAMF7 was found to have increased expression on M1 macrophages in a murine model with Pseudomonas aeruginosa keratitis [11], which, in turn, promoted polarization of M2 through signal transducer and activators of transcription 6 (STAT6) activation and alleviated corneal inflammation [11]. The checks and balances between the differentiated states of the macrophages may also play a role in *Acanthamoeba* infection. However, the polarization states of macrophage, the expression level of SLAMF7, and the transcription factor STAT6 remain unclear in AK.

In this study, we used bulk RNA sequencing to illustrate the mRNA expression and enriched signaling pathways of corneas in AK patients, which may contribute to unveiling the immune condition between *Acanthamoeba* and host cornea. At the same time, we investigated the expression level of the SLAMF7/STAT6 pathway in the late stage of AK. The activation of SLAMF7/STAT6 signaling may affect immunological competence and tissue repair in AK. These findings may provide evidence and novel targets for the immunotherapy of AK.

## 2. Materials and Methods

### 2.1. Patients Enrolled

This study was conducted at the Beijing Tongren Hospital between September 2021 and May 2022 with the approval of the Medical Ethics Committee of Beijing Tongren Hospital (TRECKY2021-024), Beijing, China. The cornea tissues were from three AK patients and three donors from the local eye bank (Beijing Tongren Eye bank, Beijing, China). Because of an ethical problem, only corneal tissue in the advanced or late stage of AK cases could be collected. Based on Jiang’s staging criteria for AK, the inclusion of late-stage AK in this study was as follows: (1) typical clinical manifestations and at least one positive laboratory test (corneal scraping or cultures for *Acanthamoeba*); (2) presence of a deep stromal ulcer (diameter > 4 mm), a pale or yellowish-white purulent infiltration, and obvious anterior chamber hypopyon. At the same time, these participants with a history of ocular infection, ocular inflammation, ocular trauma or eye surgery, mixed ocular infections, cancer, and chronic infections such as Hepatitis B, C, and HIV were excluded. All participants were informed of the goals of the study and their written informed consent was obtained in accordance with the declaration of Helsinki. Patient information was recorded using a standard protocol, which included demographics, time to diagnosis from symptom onset, risk factors, a history of steroid use, preoperative and postoperative visual acuity of the affected eye, and surgical modality. Risk factors for AK include contact lens wear, ocular trauma, or exposure to potentially contaminated water. For all subjects, slit lamp, and in vivo confocal microscopy (IVCM) images were obtained. Slit lamp images were taken per eye at 10× times magnification using a calibrated Haag-Streit BX900 slit-lamp bio-microscope (Haag Streit AG, Bern, Switzerland). In addition, the confocal microscope HRT3 (Heidelberg Engineering, Heidelberg, Germany) was used for detecting *Acanthamoeba* trophozoite and cysts, and inflammatory cells with 800 magnifications. Laboratory examinations for AK included corneal scrapings, optical microscopic observation after Giemsa staining, and cultures on non-nutrient agar plates overlaid with Escherichia coli, performed in the Department of Ocular Microbiology at the Beijing Institute of Ophthalmology.

### 2.2. mRNA Extraction and Sequencing

Three AK patients received corneal transplantation. Half of each patient’s corneal button was used for RNA extraction. For two of the three patients, the other half of the corneal button was used for a histopathology examination. For the third patient, a quarter of the other half of the corneal button was used in Western blot, and the other quarter of that was used in quantitative real-time PCR (qRT-PCR). Total RNA was extracted and purified from cornea tissue using TRIzol reagent (Invitrogen, Carlsbad, CA, USA) following the manufacturer’s procedure. The RNA amount and purity of each sample were quantified using NanoDrop ND-1000 (NanoDrop, Wilmington, DE, USA). The RNA integrity was assessed by Bioanalyzer 2100 (Agilent, Santa Clara, CA, USA) with RIN number > 7.0 and confirmed by electrophoresis with denaturing agarose gel. Poly-A mRNA was purified from 1 μg total RNA using Dynabeads Oligo (dT)25-61005 (Thermo Fisher, Carlsbad, CA, USA) using two rounds of purification. Then, the poly-A mRNA was fragmented into small pieces using Magnesium RNA Fragmentation Module (NEB, cat. e6150, Ipswich, MA, USA) at 94 °C 5 min. Then, the cleaved RNA fragments were reverse transcribed to create the cDNA by SuperScript™ II Reverse Transcriptase (Invitrogen, cat. 1896649, USA), which were next used to synthesize U-labeled second-stranded DNAs with Escherichia coli DNA polymerase I (NEB, cat.m0209, USA), RNase H (NEB, cat.m0297, USA), and dUTP Solution (Thermo Fisher, cat. R0133, USA). An A-base was then added to the blunt ends of each strand, preparing them for ligation to the indexed adapters. Each adapter contains a T-base overhang for ligating the adapter to the A-tailed fragmented DNA. Single- or dual-index adapters were ligated to the fragments and size selection was performed with AMPureXP beads. After the heat-labile UDG enzyme (NEB, cat.m0280, USA) treatment of the U-labeled second-stranded DNAs, the ligated products were amplified with PCR. The average insert size for the final cDNA library was 300 ± 50 bp. At last, we performed the 2 × 150 bp paired-end sequencing (PE150) on an illumina Novaseq™ 6000.

### 2.3. Differentially Expressed Gene Analysis

Fastp software (https://github.com/OpenGene/fastp, accessed on 1 May 2022) was used to remove the reads that contained adaptor contamination, low-quality bases, and undetermined bases with default parameters. Then, sequence quality was also verified using fastp. HISAT2 (https://ccb.jhu.edu/software/hisat2, accessed on 1 May 2022) was used to map reads to the reference genome of Homo sapiens GRCh38. The mapped reads of each sample were assembled using StringTie (https://ccb.jhu.edu/software/stringtie, accessed on 1 May 2022) with default parameters. Then, all transcriptomes from all samples were merged to reconstruct a comprehensive transcriptome using gffcompare (https://github.com/gpertea/gffcompare/, accessed on 1 May 2022). After the final transcriptome generation, StringTie was used to determine the expression level for mRNAs by calculating FPKM. The differentially expressed mRNAs were selected with fold change > 4 or fold change < −4 and with *p* value < 0.05 by R package DESeq2 (https://github.com/mikelove/DESeq2, accessed on 10 May 2022).

### 2.4. Pathway Enrichment Analysis

To better understand the function of differentially expressed genes, Gene Ontology (GO) and the Kyoto Encyclopedia of Genes and Genomes (KEGG) were performed on the obtained differential genes. Since GO term has three independent aspects of biological process (BP), cellular component (CC), and molecular function (MF), clustering results were obtained based on the semantic similarity of GO terms. Then, KEGG and GO term gene set enrichment analysis (GSEA) was conducted using clusterProfiler. Normalized enrichment score (|NES| > 1), nominal *p* < 0.05, and false discovery rate (FDR) < 0.25 were considered statistically significant. The raw counts were loaded into R 4.2.1 (R Foundation for Statistical Computing, Vienna, Austria) for statistical analysis and the ridge plot was used for data representation.

### 2.5. Immune Infiltration Analysis

Based on Chen et al. method of CIBERSORT, the RNA content of all tissues was analyzed [12]. Under LM22 as the signature gene file and permutation = 1000, the 22 immune cell profiles were reconstructed. The sum of all proportions of immune cell fractions was normalized to 1 in each sample and ggplot2 (version 3.3.6; https://ggplot2.tidyverse.org/, accessed on 1 July 2022) was used to generate a stacked bar plot for better visualization. Further, the 4 aggregated immune cell types, including total lymphocytes, total dendritic cells, total macrophage, and total mast cell were calculated, following the method of Li et al. [13].

### 2.6. Histopathology Examinations

The corneal button was fixed in 4% paraformaldehyde (PFA) overnight and embedded in paraffin. Then, it was sectioned with a thickness of 4 μm, and slices were dewaxed and hydrated. Hematoxylin and eosin (HE) staining was conducted according to routine protocols. The slices for immunohistochemical staining were repaired with EDTA buffer. Endogenous peroxide activity was blocked by incubation of the slides with 3% H_2_O_2_ for 20 min. After being permeabilized (0.5% Triton X-100 in PBS) and blocked (10% Normal donkey serum and 0.5% Triton in PBS), the rabbit anti-human CD8a (1:800; Cell Signaling Technology, Danvers, MA, USA, #98941), CD4 (1:1000; Abcam, Boston, MA, USA, #ab183685), SLAMF7 (1:2000; Abcam, #ab32520), and STAT6 antibodies (1:200; Abcam, #ab230945) were applied overnight at 4 °C. The sections were washed and incubated with HRP-conjugated goat anti-rabbit IgG (1: 2000; Abcam, #ab205718) for 1 h at room temperature. They were photographed with a microscope equipped with a digital camera (Olympus BX-51, Olympus, Tokyo, Japan) and the cells with brown-stained cytoplasm were considered positive.

### 2.7. Western Analysis

Equal amounts of proteins were electrophoresed on 10% PAGE gels and transferred to a polyvinylidene fluoride membrane with a wet transfer system. The membranes were blocked with 5% skim milk for 1 h. After washing with PBS plus 0.05% Tween 20 (PBST), the membrane was incubated with primary antibodies overnight at 4 °C. In addition to the anti-human SLAMF7 and STAT6 antibodies described above, the rabbit anti-human phospho-stat6 antibody (1:1000; Cell Signaling Technology, #9361) and GAPDH antibody (1:2000; Proteintech, Rosemont, IL, USA, #1E6D9) were also used for Western analysis. The following day, the same secondary antibody as described in the aforementioned histopathology examinations was performed for 1 h at room temperature. Membranes were visualized by chemiluminescence using an enhanced chemiluminescence substrate.

### 2.8. Quantitative Real-Time PCR

Total RNA of the cornea was extracted using Tissue Total RNA Isolation Kit V2 (Vazyme Medical Technology, Nanjing, China). RNA was reverse transcribed into cDNA using the qPCR RT Master Mix Kit (FSQ-301, Toyobo, Japan). qRT-PCR was performed using the Taq Pro Universal SYBR qPCR Master Mix (Vazyme Medical Technology, Nanjing, China) and conducted on ABI 7500 System. All experiments were repeated at least three times, and *p* < 0.05 indicated a statistically significant difference. Relative expression levels were calculated using the comparative threshold cycle (Ct) method. Human β-actin expression acted as an endogenous reference to normalize relative gene expression. Primer sets used in qRT-PCR are listed in Table S1. 

### 2.9. Statistical Analysis

The data of qRT-PCR are presented as the mean ± standard error of the mean (SEM) according to statistical analysis. Statistical comparisons were analyzed using an unpaired Student’s *t*-test by graphing software. Correlation between RNA-seq and qRT-PCR was calculated by linear regression and Pearson correlation. *p* < 0.05 indicated statistical significance.

## 3. Results

### 3.1. Clinical Manifestations

Three patients (two females and one male) with AK were recruited. Diagnosis was based on the clinical manifestations and laboratory findings. The results of corneal scraping, culture, and IVCM of these patients were all positive for AK. The mean age of patients with AK (44.0 ± 17.0 years) was comparable to that of the control (46.0 ± 13.6 years, *p* = 0.903). Their major risk factors included agricultural injury and orthokeratology lens wearing. The mean time between the first clinical symptom and the definitive diagnosis was 32.7 ± 4.2 days. Figure S1 provides the preoperative slit-lamp photograph, and the mean ulcer size was 7.2 ± 1.6 mm. Two patients had undergone penetrating keratoplasty (PK) and the other received deep anterior lamellar keratoplasty (DALK). Visual improvement was observed in these three cases after surgery (Table S2).

### 3.2. Assessment of Sequencing Data Quality

**Figure 1:**
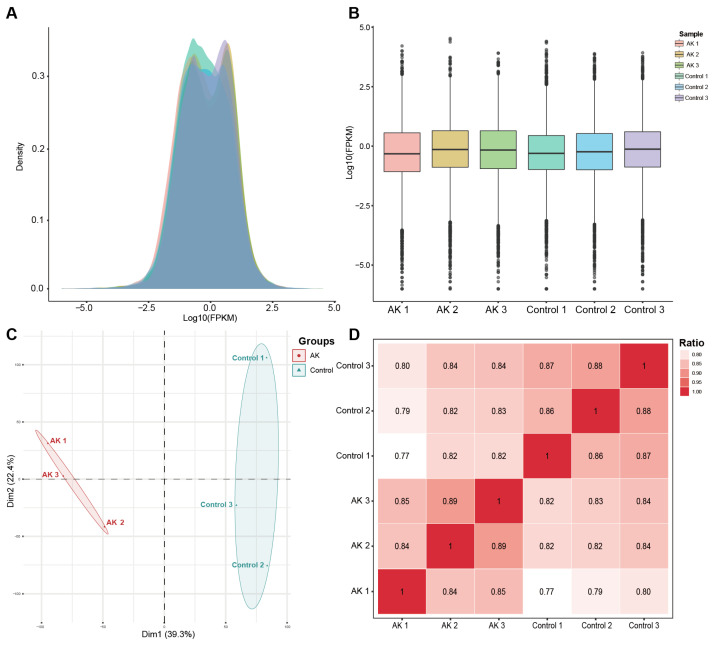
RNA sequencing data quality control and consistency tests. (**A**) FPKM density distribution of the sequencing data. The *x*-axis represents the log10 (FPKM) value of the gene and the *y*-axis represents the distribution density of the genes with corresponding expressions. (**B**) Box plot of the FPKM density distribution. (**C**) Principal component analysis of the sequencing samples. (**D**) Correlation test of the sequencing samples.

The expected number of fragments per kilobase of transcript sequence per million reads mapped (FPKM) density and saturation distribution at the transcriptome level in the two groups of sequencing samples were detected. The statistical results of the expression characteristics of all the genes in each sample are shown in Figure 1A,B. The results of the principal component analysis and sample correlation tests showed that there was clear heterogeneity among the two groups of six sequencing samples, as shown in Figure 1C,D. The above information showed that the high-throughput sequencing data were of high quality and met the standards for further analysis.

### 3.3. Significant Differentially Expressed Genes Associated with Acanthamoeba Keratitis

**Figure 2:**
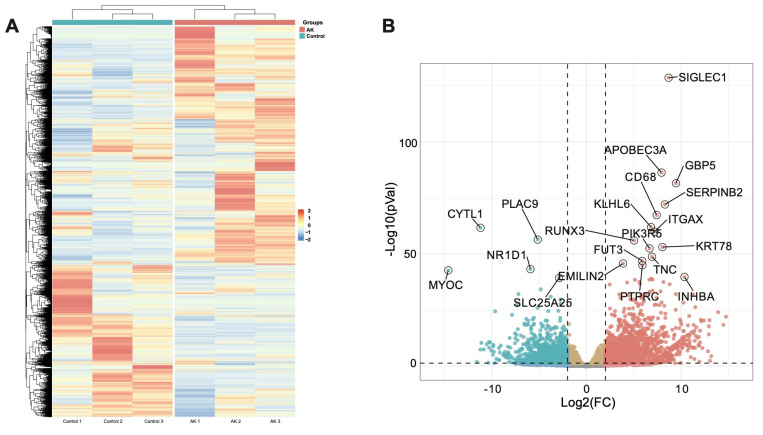
Results of the DEGs between AK group and control group. (**A**) A heatmap of each significant DEG in different samples. (**B**) Volcano plot of the significant DEGs between two groups. The *x*-axis represents the log2 fold change and the *y*-axis represents −log10 (*p* value) of each significant DEG.

Based on the alignment with the human reference genome, a total of 18,249 genes transcripts were identified. By comparing the gene expression profiles of the AK cases and normal controls, based on the threshold criterion of log2 fold change > 4 and *p* < 0.05 significance, 2668 differentially expressed genes were screened, including 1477 upregulated genes and 1191 downregulated genes (Figure 2A). Excluding the factor of zero value, the sialic-acid-binding immunoglobulin-type lectins-1, CD169 (SIGLEC1) gene, which was linked to type I interferons [14], increased eightfold in the AK group and was the most significantly upregulated gene. SIGLEC1 was a sialic-acid-binding cell-surface protein, exclusively expressed on monocytes and macrophages. When the expression of SIGLEC1 was elevated, it was linked to type I interferons and, to a lesser degree, other activator stimuli, such as LPS [14]. Among the 1191 significantly downregulated genes, expression of the CYTL1 (cytokine-like protein 1) gene in the AK group was the lowest. The expression of this gene was approximately 11 times lower in the AK group than in the control group. The previous literature had reported that CYTL1 was a small widely expressed secreted protein, which closely related to CCL2, and the expression was also downregulated in the squamous cell carcinoma of the lung [15]. The top 20 significantly altered genes were annotated in a volcano plot (Figure 2B).

### 3.4. Significantly Enriched Pathways Associated with Acanthamoeba Keratitis

**Figure 3:**
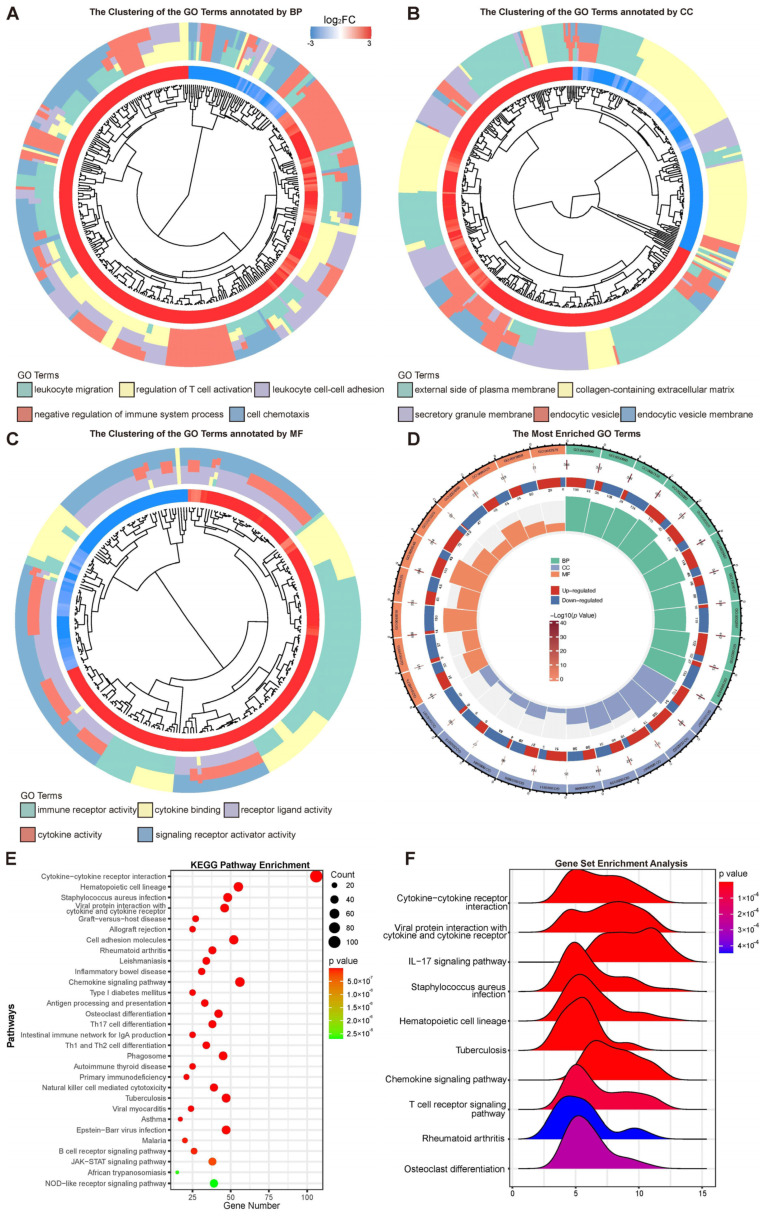
Signal-transduction-related pathways enriched. (**A**–**C**) Clustering results based on semantic similarity of GO terms annotated by biological process (BP), cellular component (CC), and molecular function (MF); (**D**) significant signal-transduction-related pathways were enriched by GO analysis. From the outer circle to the inner circle: the top 10 pathways annotated by BP, CC, and MF; the result of −log10 (*p* value) corresponding to the pathway; the number of genes upregulated or downregulated in this pathway; the total number of genes associated in that pathway; (**E**) top 30 significant signal-transduction-related pathways were enriched by KEGG analysis. The *x*-axis represents gene number and the *y*-axis represents the name of each pathway. The color scale indicates the significant level of pathway enrichment; (**F**) the ridge plot depicted top 10 signaling pathways enriched by the gene-set enrichment analysis.

Based on the screened DEGs and the method of GO analysis, the significantly enriched signal transduction pathways related to the pathogenesis and immunomodulatory of AK in humans were identified. Compared with the control group, the AK group revealed 1099 significantly different signaling pathways by GO analysis. To further understand the results of signaling pathways enriched by GO analysis, the GO terms were clustered based on semantic similarity. Figure 3A revealed the cluster of pathways annotated by biological process (BP) category of GO terms, contained leukocyte migration, regulation of T-cell activation, leukocyte cell–cell adhesion, negative regulation of the immune system process, and cell chemotaxis. When the classification was based on cellular component (CC) category, the cluster contained the external side of the plasma membrane, collagen-containing extracellular matrix, secretory granule membrane, endocytic vesicle, and endocytic vesicle membrane (Figure 3B). In addition, the classification with molecular function (MF) provided the cluster of pathways including immune receptor activity, cytokine binding, receptor ligand activity, cytokine activity, and signaling receptor activator activity (Figure 3C). Each top 10 GO pathway of BP, CC, and MF is shown in the Figure 3D. The −log10(*p* value), the number of up- and downregulated genes, and the total number of genes in the pathway are also sketched out in Figure 3D. KEGG pathway analysis of DEGs showed, in total, 58 pathways were significantly enriched (*p* < 0.05). KEGG pathway enrichment analysis suggested that the top 30 pathways related with DEGs were cytokine–cytokine receptor interaction (Figure 3E). Similar GO terms and KEGG pathways were observed via GSEA analysis (Figure 3F). Cytokine–cytokine receptor interaction was determined as the most likely difference between AK and control group. In addition, the enriched terms also included viral protein interaction with cytokine and cytokine receptor, IL-17 signaling pathway, Staphylococcus aureus infection, and hematopoietic cell lineage. A previous study reported IL-17A production plays an important role against Acanthamoeba infection. The mice with IL-17A deficiency had significantly increased corneal AK pathology [16].

### 3.5. Distribution of the Immune Cells in Acanthamoeba Keratitis

**Figure 4:**
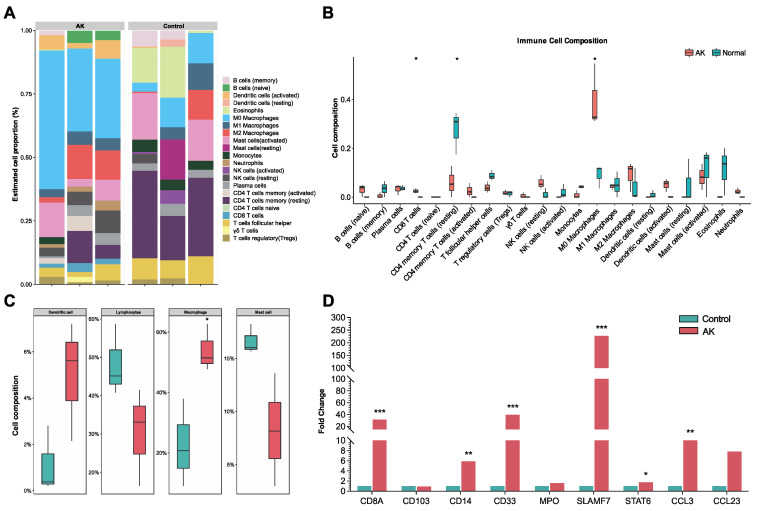
Distribution of the immune cells (22 types of immune cells) in cornea between AK and control group by CIBERSORT algorithm. (**A**) The proportions of immune cells in each cornea. (**B**) Comparison of immune cell proportions between AK and control group. (**C**) Comparison of 4 aggregated immune cell types between AK and control group. (**D**) Expression patterns of immune-cell-related gene module were shown in AK and control group. * *p* < 0.05, ** *p* < 0.01, *** *p* < 0.001.

CIBERSORT algorithm provided the abundance of immune cells in each cornea (Figure 4A). RNA-seq data were pooled and performed group comparisons. Figure 4B showed significantly higher immunocyte infiltration degrees of M0 macrophages and CD8 T cells and significantly lower immunocyte infiltration degrees of CD4 memory resting T cells in AK group. The immunocyte infiltration degrees of M2 macrophages were also increased but were not statistically significant in the level of *p* < 0.05. There was no significant intergroup difference in neutrophil, NK cells, B cells, and dendritic cells. A further aggregated version based on cell type provided the results that the macrophage was significantly increased in AK group (Figure 4C). The dendritic cell was also increased, and lymphocytes and mast cells decreased in patients with AK. However, those differences were not statistically significant. Based on the results above, we revisit the cell marker of M0 (CD33 and CD14), M1 (IL-1β and CD86), and M2 (CD163 and CD206), associated regulators, and chemokine in the results of DGEs. The outcomes revealed that macrophage-associated markers were significantly higher, especially the marker of M2 (*p* < 0.001). M2-associated regulators (SLAMF7 and STAT6) and the chemokine CCL3 were significantly increased in the AK group (*p* < 0.01). However, the difference in chemokine CCL23 did not reach significance (*p* = 0.08) (Figure 4D).

### 3.6. Validation of Expression Genes in Acanthamoeba Keratitis

**Figure 5:**
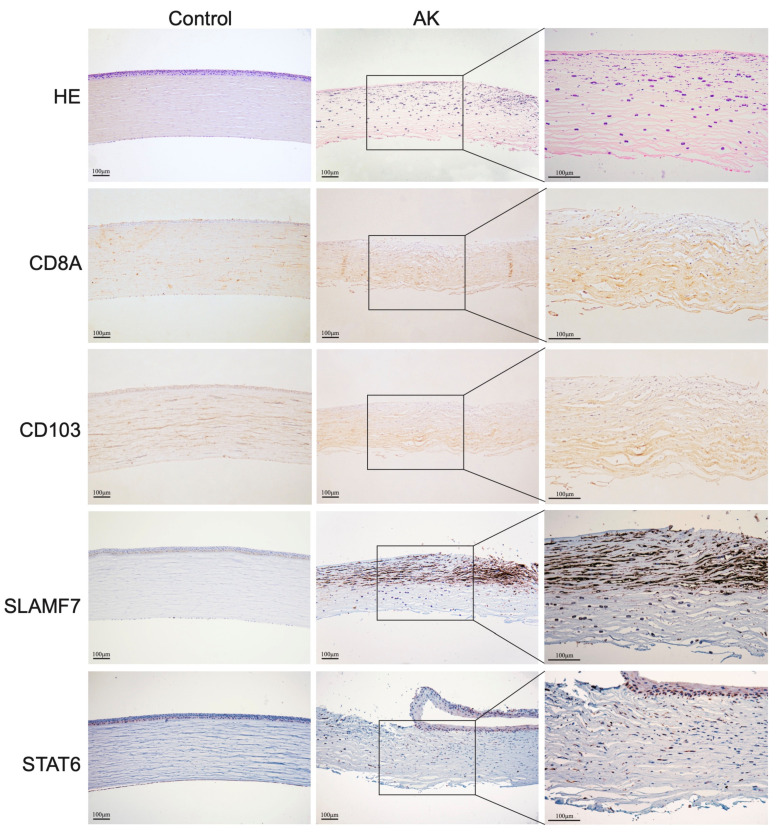
Representative images of CD8a, CD4, SLAMF7, and STAT6 immunohistochemistry staining in the different groups. Scale bars: 100 μm.

**Figure 6:**
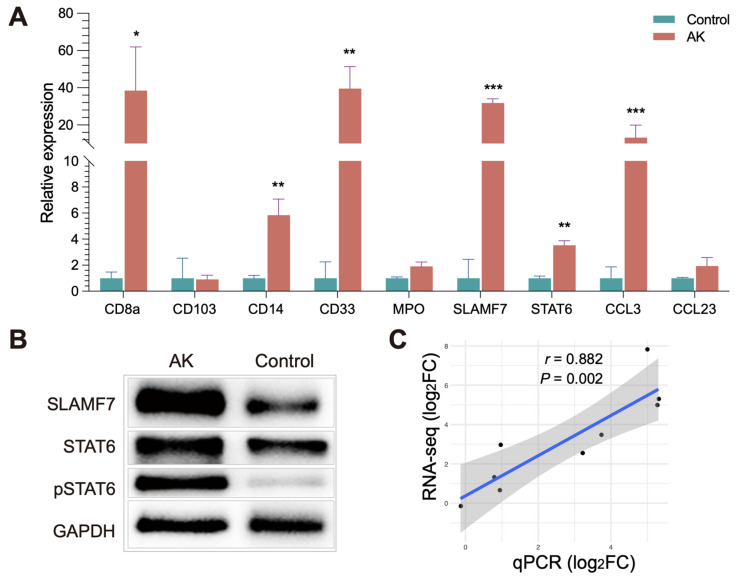
RT-qPCR and Western blot analysis confirming the validity of the RNA-seq results. (**A**) The relative expression levels of the DEGs between the AK and control groups were analyzed by RT-qPCR. * *p* < 0.05, ** *p* < 0.01, *** *p* < 0.001. (**B**) The protein levels of SLAMF7, pSTAT6, and STAT6 in cornea. (**C**) Correlation of the gene expression levels between RT-qPCR and bioinformatics data.

Furthermore, to verify the bioinformatics results, seven DEGs were validated for selected genes by immunohistochemistry, quantitative real-time PCR, and Western blot and a similar trend of expression was obtained. CD8a staining was weakly positive by immunohistochemistry and no significant difference was found between groups. Immunohistochemistry also did not reveal clear differences in the expression levels of CD4. Comparing the present immunohistochemical staining, it is evident that the expression of SLAMF7 and STAT6 have significant differences between groups (Figure 5). The bioinformatics results were also validated for selected genes by quantitative real-time PCR (Figure 6A), and a similar trend of expression was obtained. Western blot also showed the level of phosphor-STAT6, which was significantly increased compared with control (Figure 6B). In general, the results also showed good consistency of the qPCR results and RNA sequencing results (Figure 6C), demonstrating reliability of the RNA-seq data (r = 0.882, *p* = 0.002).

## 4. Discussion

AK is a rare disease with unfavorable therapeutic efficacy and dismal prognosis, which could occur in normal individuals [17]. To date, the process of the immune response in the cornea of AK has been slightly revealed. To our knowledge, this is the first study to make bulk RNA sequencing of corneas in AK patients and analyze the immune state. In the current study, we performed a comprehensive evaluation of the immune landscape of AK and found that only several immune cells (M0 macrophage and CD8 T cells) had a significantly increased infiltration. However, most immune cells lacked significant infiltrate in the cornea of AK patients, compared with completely healthy controls. The findings herein suggested that escape from recognition by immune effector cells might appear in late-stage AK and ultimately lead to an extremely poor prognosis.

Previous studies had suggested this point. The clinicopathologic report found the macrophage and neutrophils presented in regions with cysts, but distribution of CD4 or CD8 T cells did not directly associate with *Acanthamoeba* parasites. NK cells were absent in cornea of AK-infected individuals in the report [18]. Another clinicopathologic study also found the *Acanthamoeba* cysts grouped in a zone without noticeable inflammation [9]. Compared with our study, they mentioned the infiltration of neutrophils, the origin of which is probably from the granulomatous retro-corneal membrane in their sample. Typically, the neutrophils are identified as the first responders to acute infections. In the current study, the samples were taken from the late-stage AK patients and HE staining showed no additional retro-corneal membrane.

The innate immune system is responsible for immediate host defense against pathogens upon infection. Former research in fungal keratitis indicated that inflammatory cytokines, IL-1β, IL-6, TNF- α, as well as Wnt, Hippo, and cGMP-PKG signaling pathways were significantly associated with the disease [19]. Transcriptome analysis showed dominated innate immune response and lower levels of adaptive immune pathways in both late-stage bacterial and late-stage fungal keratitis [20]. Meanwhile, in AK, T cells and B cells were not found around *Acanthamoeba*-infected cornea regions and serum antibodies are not protective against this infection, as recrudescence also occurs [18,21], suggesting that adaptive immune response fails to protect the host and innate immune response is vital for disease resolution. As the depletion of conjunctival macrophages led to exacerbated *Acanthamoeba* infection and increased number of neutrophils were found to accumulate in *Acanthamoeba*-infected corneas [10,22], macrophages and neutrophils were believed to be two elements in the innate immune system important in *Acanthamoeba* infection [23]. Both cells were shown to kill *Acanthamoeba* trophozoites in vitro [24,25]. In vivo, although cysts are not chemotactic towards macrophages and neutrophils, their lysates can be recognized and, thus, be phagocytosed by macrophages and be killed by neutrophils via myeloperoxidase [26,27,28]. Interestingly, unlike neutrophils, macrophages were shown to maintain at the infection site, demonstrating their role in mounting immune response, as well as tissue repair [18,29,30]. The state of pathway may provide the clue. In our study, lots of immune signaling pathways were activated, such as the leukocyte migration, leukocyte cell–cell adhesion, and immune receptor activity. At the same time, many pathways about immunoregulation were activated to inhibit the immune response, such as the regulation of T cell activation and the negative regulation of the immune system process. Several immune pathways, which most significantly increase in expression, have been identified as being of value in the defense against *Acanthamoeba* infection, such as the IL-17 signaling pathway and Th17 cell differentiation [16,31]. The cross-talk among immune signaling pathways resulted in the current immune status.

We found part of the landscape of immune condition in human late-stage AK, where dendritic cells and macrophages increased, while lymphocytes decreased. As antigen-presenting cells, dendritic cells are crucial in both innate and acquired immune responses. The increased activated dendritic cells are in line with the concept that microbial molecules could induce the maturation of dendritic cells, while the decreased lymphocytes suggested that signaling pathways between dendritic cells and lymphocytes may be hampered in late-stage AK [29]. M0 macrophages were significantly increased in the AK group compared to the control. Previous studies have found that *Acanthamoeba* can activate proinflammatory M1 macrophage to produce IL-12 and IL-6 via TLR4-MyD88 pathway [30]. In our study, transcriptome data of late-stage AK showed that the amount of M1 macrophage had no difference between AK and control groups, and M2 macrophages are increased in the AK group. SLAMF7, the regulator associated with M2 macrophages, was significantly increased in the AK group, suggesting its association with M2 polarization. Changes in SLAMF7 and STAT6 in AK patients were also observed by immunohistochemistry. Figure 6A,B show that SLAMF7 and STAT6 were abundant in AK groups, mainly expressed in epithelial and anterior stromal cells. The activation of SLAMF7/STAT6 signaling pathway may offer direction about where to intervene in AK. The treatment of AK should gradually change from the traditional pharmacological treatment to immunotherapy. Therefore, excavating novel therapeutic targets for AK is significant. The present study provided clues for further study and data source.

Still, some limitations are present in this study. For example, in that lack of corneas from AK patients resulted in the small number of specimens recruited for analysis. Because bulk RNA sequencing is subject to having less accurate analysis to reveal the immune condition, single-cell sequencing should be carried out in the future and the results should be compared to other types of keratitis. On the other hand, although the function of SLAMF7/STAT6 pathway had been validated in *P. aeruginosa* keratitis and the expression of pathway had been observed in AK, further experimental validation is required to verify the effect and major target of SLAMF7/STAT6 pathway.

## 5. Conclusions

To our knowledge, this is the first transcriptome analysis of AK, and we initially shape the immune landscape of AK. M0 macrophage is the principal immune cell, but most immune cells infiltrated lower in late-stage AK. We also preliminarily explored the role of SLAMF7/STAT6 pathway in AK, which was considered to perturb the polarization direction of macrophage. Taken together, these observations suggested there might be an inhibited immune response in AK and could provide potential directions for further treatment.

## Data Availability

Not applicable.

## Supplementary Materials

The following supporting information can be downloaded at: https://www.mdpi.com/article/10.3390/microorganisms11020365/s1, Figure S1: The slit-lamp and in vivo confocal microscopy images of three recruited patients with *Acanthamoeba* keratitis; Table S1: Nucleotide sequences of human primers for RT-qPCR; Table S2: Demographics and clinical characteristics of patients with *Acanthamoeba* keratitis.
