# Supplementary material for: SLAMF7/STAT6 Pathway Inhibits Innate Immune Response in Late-Stage Human Acanthamoeba Keratitis: A Comparative Transcriptome Analysis
Source: Microorganisms. 2023 Feb 1;11(2):365. doi: 10.3390/microorganisms11020365 (PMC9961001; doi:10.3390/microorganisms11020365)
Supplement: Supplementary file 1 [file microorganisms-11-00365-s001.zip › microorganisms-2159209-supplementary.pdf]

Table S1. Nucleotide sequences of human primers for RT-qPCR.

Name	Forward	Reverse
CD8a	CCCTTTACTGCAACCACAGG	GTCTCCCGATTTGACCACAG
CD103	ACCATTCTTTGCCTATCATCATTAAAG	CTCCATGCTGCTCTAGATCATC
CD14	GACCTAAAGATAACCGGCACC	GCAATGCTCAGTACCTTGAGG
CD33	GGTGTGACTACGGAGAGAACC	GGTAGGGTGGGTGTCATTCC
MPO	CCGGGATGGTGATCGGTTTT	CAGATGATCCGGGGCAATGA
CCL3	GCAACCAGTTCTCTGCATCA	TGGCTGCTCGTCTCAAAGTA
CCL23	CATCTCCTACACCCCACGAAG	GGGTTGGCACAGAAACGTC
β-Actin(human)	GATTACTGCTCTGGCTCCTAGC	GACTCATCGTACTCCTGCTTGC

Table S2. Demographics and Clinical Characteristics of patients with *Acanthamoeba* keratitis.

Parameter	Patients 1	Patients 2	Patients 3
**Subject (Age, gender)**	57y, FM	55y, FM	20y, M
**Onset time (days)**	37	34	27
**Risk Factor**	Unknown	Agricultural injury, Tap water exposure	Orthokeratology, Tap water exposure
**Preoperative/ Postoperative visual acuity**	HM / 0.01	CF / 0.01	0.01 / 0.3
**Steroids used**	Y	N	N
**Ulcer size** (mm)	9	7	5
**Scraping (+/-)**	+	+	+
**Culture (+/-)**	+	+	+
**IVCM (+/-)**	+	+	+
Surgical Interventions	PK	PK	DALK

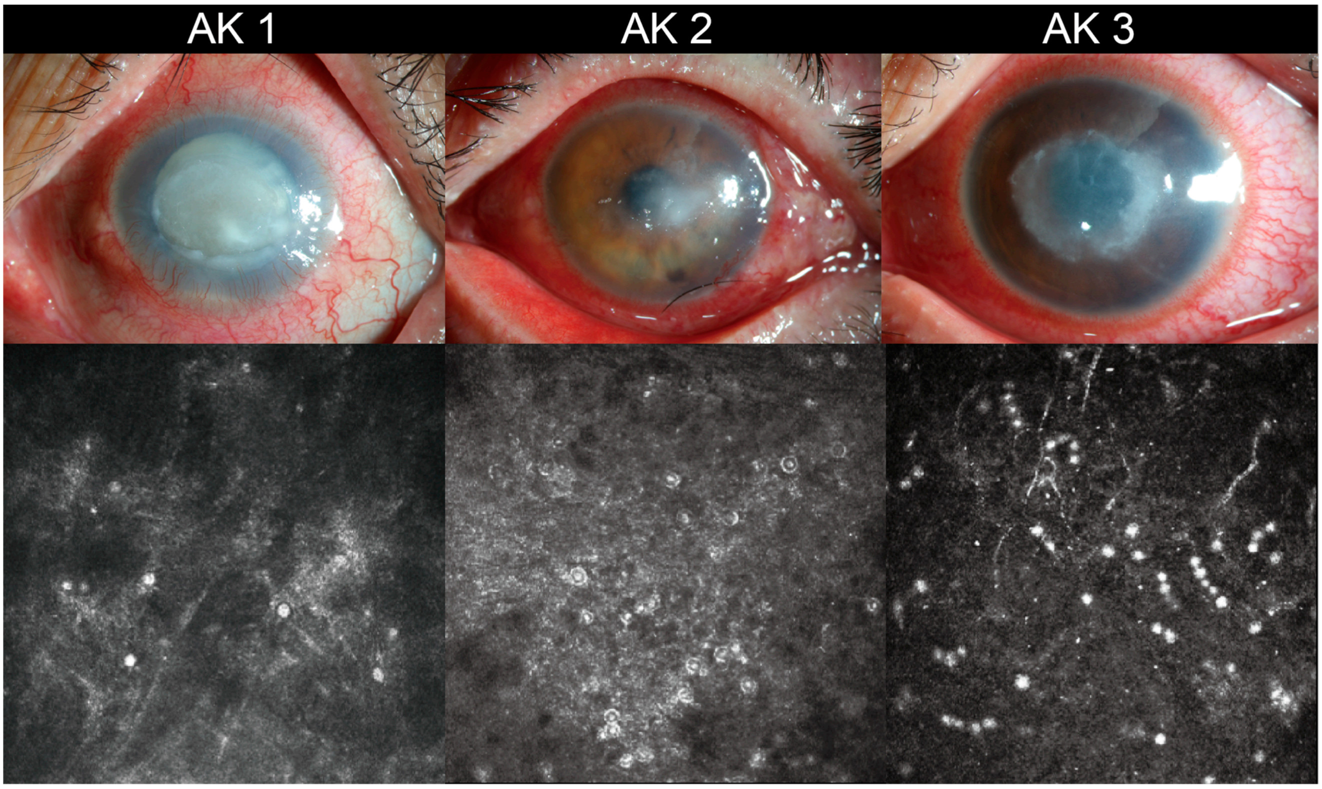

Figure S1. The slit-lamp and in vivo confocal microscopy images of three recruited patients with *Acanthamoeba* keratitis.
